# Impacts of PAH accumulation on reproductive hormones, indices of oxidative stress and BPDE-albumin adduct in women with recurrent pregnancy loss

**DOI:** 10.1007/s43188-023-00181-5

**Published:** 2023-05-20

**Authors:** Amany El-Sikaily, Mohamed Helal, Augusta Chinyere Nsonwu-Anyanwu, Hossam Azab, Neveen Abd ElMoneim, Eman Othman Salem Farahat, Aziza Saad

**Affiliations:** 1 National Institute of Oceanography and Fisheries (NIOF), Cairo, Egypt; 2 Department of Clinical Chemistry and Immunology, University of Calabar, Calabar, Nigeria; 3 Obstetrics and Gynecology Department, Faculty of Medicine, Alexandria University, Alexandria, Egypt; 4 Applied Medical Chemistry, Medical Research Institute, Alexandria University, Alexandria, Egypt

**Keywords:** Antioxidants, Polycyclic aromatic hydrocarbon, Lipid peroxidation, Mussels, Pregnancy

## Abstract

Chronic exposure to Poly aromatic hydrocarbons (PAHs) may be associated with adverse pregnancy outcomes. Disruption of hormonal and redox balance by toxic PAH metabolites may interfere with successful pregnancy leading to miscarriage. The association of exposure to PAH contaminated mussel via the dietary route with perturbations in reproductive hormones, biomarkers of oxidative stress, and PAH metabolites were assessed in women with recurrent pregnancy loss (RPL). Furthermore, an analysis of the concentration of PAHs in environmentally relevant bivalve animals was performed to preliminary get insights into the levels of these pollutants in the environment. Seventy-six women (20–35 years) were categorized into 18 fertile women without RPL (control), and Groups I, II, and III comprising 24, 18, and 16 women with RPL (2, 3, and > 3 abortions respectively) were studied. Whole blood samples were collected for the estimation of malondialdehyde (MDA), catalase, reduced glutathione (GSH), glutathione-S-transferase (GST), progesterone (P4), follicle-stimulating hormone (FSH), benzo[a]pyren-7,8-dihydrodiol-9,10-epoxide-albumin adduct (BPDE-albumin) and urine for α-naphthol and β-naphthol. Two species of mussel *Donax trunculus* and *Andar aduloii* samples were collected for the estimation of 16 priority PAHs. The concentration of PAHs exceeding the maximum limits was observed in the two species of mussels studied. Higher levels of BPDE-albumin, MDA, GST, α and β-naphthol and lower GSH, catalase, FSH, and P4 were observed in women with RPL (Groups I-III) compared to controls (*p* =  < 0.001). Negative associations were observed between BPDE-albumin and catalase (r = − 0.276, *p* = 0.036), and GSH (r = − 0.331, *p* = − 0.011) only in women with RPL. Collectively, our findings indicate a possible association of chronic PAH accumulation with recurrent pregnancy loss in women.

**Graphical abstract:**

High PAH exposure in pregnant women is associated with 10-epoxide-albumin adduct formation and high MDA levels in their sera. On the other hand, PAH exposure in those women led to a decrease in their GSH, catalase, P4, and FSH sera levels. These findings indicate that PAH exposure can exert different physiological effects in pregnant women leading to a high level of abortion in those women.
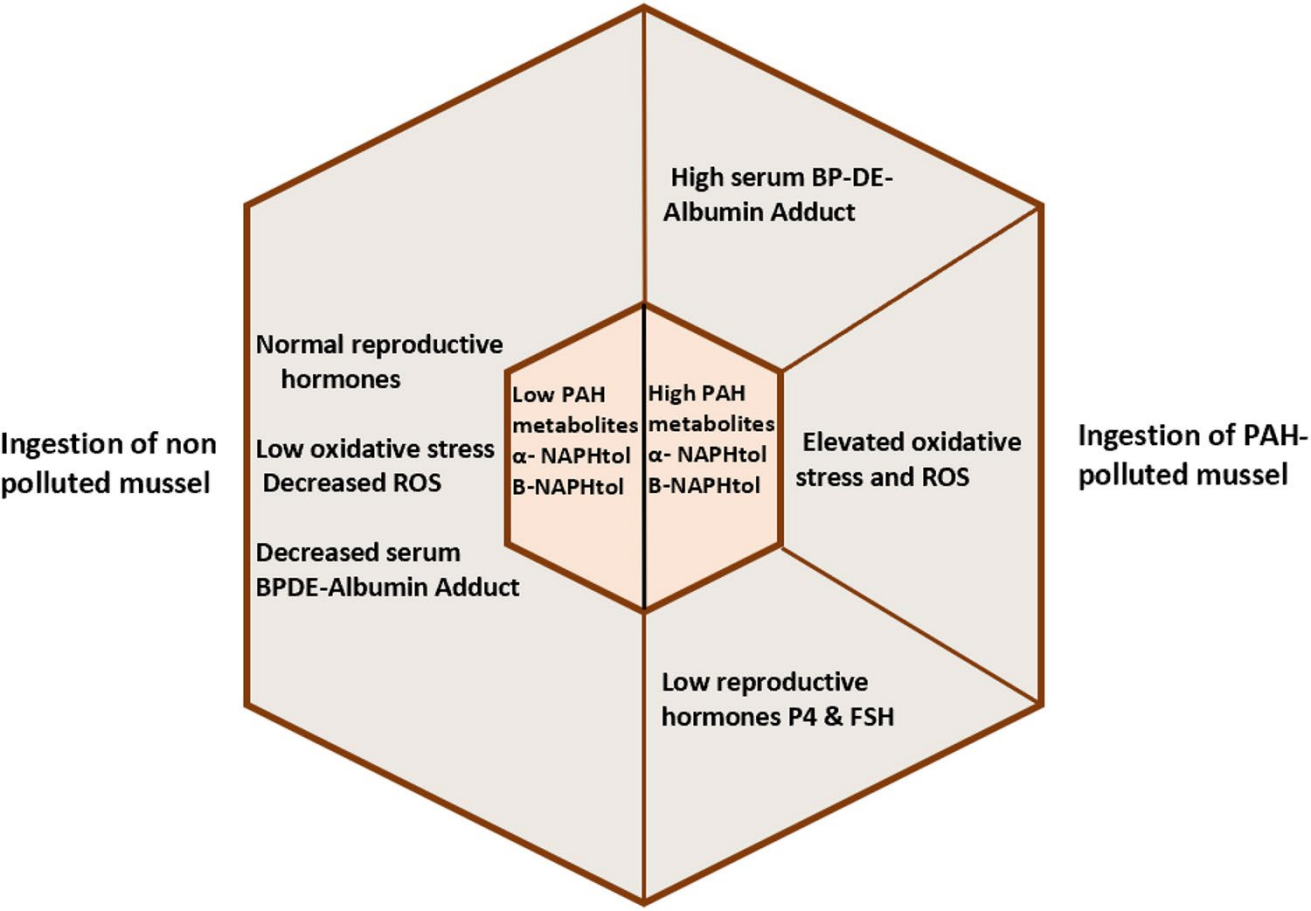

## Introduction

Myriads of public health effects including carcinogenesis and multiple organ toxicities have been associated with exposure to widespread environmental toxicants including polycyclic aromatic hydrocarbons (PAHs). PAHs are released as byproducts of industrial emissions, asphalt, plastics, pesticides, medical cosmetics, tobacco smoke, exhaust fumes, and wood burning [1]. Major routes of exposure to PAHs are through inhalation of air-bone PAHs, consumption of PAH-contaminated food products, and dermal absorption [2]. Anthropogenic activity and environmental contamination with PAHs, have led to their bioaccumulation in the edible marine animals. Mussels as infiltrating animals tend to bioaccumulate PAHs in their tissues because of their limited ability to metabolize it and are therefore used as bioindicators of coastal contamination with PAH [3, 4]. Based on a combination of frequency of occurrence in nature, toxicity, and potential for human exposure, benzo[*a*]pyrene (BaP), has often been used as a marker compound or prototype of PAHs for epidemiological studies [5].

Dietary exposure to PAHs contaminated food has been associated with a wide range of multiple organ and systemic pathogenesis predominantly cancers of various organs. As lipophilic compounds, PAHs can transverse/cross-cell membranes via passive diffusion upon their ingestion or inhalation and accumulate in different organs [6]. Previous experimental studies on mammalian cells have shown evidence that the metabolic transformation of PAHs generates free radical molecules which may result in antioxidant depletion, peroxidation of lipids, protein modifications, and oxidative DNA damage [7, 8]. Increased levels of free radical molecules and decreased antioxidants have been linked to PAH exposure. In addition, reactive oxygen species (ROS) generated during PAH transformation were shown to be associated with oxidative stress induction, multiple organ toxicities, and mutagenic and carcinogenic transformations [9, 10]. Furthermore, female reproductive pathologies have been associated with PAH exposure [11–13]. Association between exposure to PAH with congenital abnormalities, growth restriction, preterm birth, reduced birth weight, length, and head circumference has been also reported by prevalence studies [13]. Epidemiological studies have documented the association of PAH exposure with endocrine abnormalities such as reduced serum progesterone (P4) levels suggesting the possible disrupting effects of this ubiquitous toxicant on the human reproductive process [11, 12, 14, 15]. Pregnancy is a critical and sensitive stage for the female and the developing fetus and exposure to environmental pollutants such as PAHs can pose a critical medical risk to the pregnant woman and the fetus [16]. Defects in fetus birth weight, size, and decreased survival were correlated to PAH exposure via inhalation (ambient air), dietary ingestion of contaminated food, and occupational exposure. These defects were also associated with PAH-DNA adduct formation [17]. Associations between PAH exposure and unfavorable pregnancy outcome have been documented, however, studies relating exposure to PAH contaminated mussel with spontaneous recurrent pregnancy loss is relatively scarce. To fill this gap in knowledge, the association of exposure to PAH-contaminated mussels with alterations in reproductive hormones, oxidative stress indices, and formation of PAH-albumin was assessed among women with recurrent pregnancy loss.

## Materials and methods

### Chemicals

Analytical grade reagents: n-hexane, methanol, acetonitrile, 1-naphthol, 2-naphthol, and β-glucuronidase from Helix pomatia (G7017) were procured from Sigma Chemicals (St Louis, USA). OxiSelect BPDE Protein Adduct ELISA Kit was purchased from Cell Biolabs, Inc. (St; 7758 Arjons Drive, San Diego, USA). The follicle stimulating hormone (FSH) and P4 levels were estimated using the Stat Fax ELISA unite (from Awareness' technology, USA) by ELISA kit from Glory Science Co. (ltd, Del Rio, USA). Test kits for the estimation of malondialdehyde (MDA), catalase, glutathione-S-transferase (GST), and reduced glutathione (GSH) were procured from Biodiagnostics Co. (29 Taahreer st; Dokki Giza, Egypt). A test kit for the estimation of creatinine was procured from Diamond Diagnostic Co. (30175 Hannover, Germany).

### Study area and design

This case study involving women with recurrent spontaneous abortions and their corresponding control counterparts was carried out in the coastal area of Alexandria using a random sampling method. Informed consent was sought and obtained from the volunteers before recruitment into the study after the ethical committee of Medical Research Institute, Alexandria University (IORG 0008812) approved the study protocol. The study was conducted in compliance with ethical principles guiding research with human subjects in accordance with the Helsinki declaration and subsequent revisions.

### Selection of subjects

The participants in this study were recruited from El-Shatby Maternity Hospital, Alexandria University, and comprised a total of 76 healthy women aged 20–35 years. The control population was made up of 18 fertile women with at least one living child after successful full-term delivery, and who had no history of recurrent spontaneous abortion. The test subjects were made up of 58 women who had experienced at least 2 successive unexplained recurrent spontaneous abortions up to 20 weeks gestational age.

A semi-structured questionnaire was administered to all participants in the study for the history of past and present ailments with an emphasis on toxoplasmosis, rubella, and herpes simplex. Women with endocrine disorders (diabetes mellitus & thyroid disorders), uterine abnormalities, hypertension, liver diseases, urinary tract insult, residents of industrial areas, and smokers were all excluded from the study.

### Sample collection

#### Urine samples

Spot urine samples (10 ml) were collected from all subjects of the study, stored at − 20 °C, and used for the estimation of PAH metabolites in urine (1-naphthol and 2-naphthol).

#### Blood samples

Whole blood samples (8 ml) were collected from all subjects of the study and distributed as follows; 3 ml was dispensed into an anticoagulant container EDTA for the estimation of glutathione and catalase enzyme activities; 5 ml was dispensed into the plain container without anticoagulant, allowed to clot and retract and then centrifuged at 4000 rpm for 10 min to extract serum for the estimation of benzo[a]pyren-7,8-dihydrodiol-9,10-epoxide-albumin adduct (BPDE-albumin), MDA, FSH and P4. The red cells were washed once with 10 volumes cold saline followed by the addition of 4 volumes of cold deionized water to get red cell pellets. The red cell storm was centrifuged at 500 g at 4 °C for 10 min. The supernatant was collected and stored at − 70 °C until the time of estimation of GST.

#### Mussel samples

Two mussel species, *Donax trunculus* and *Andar aduloii* were collected from the Mediterranean Sea “Alexandria coast” for the estimation of tissue PAH content. Mussel samples were opened raw, and the flesh was scraped out of the shell with a stainless-steel scalpel. The gills and digestive glands of the mussels were dissected out and then stored at -80 °C for determination of PAHs.

### Laboratory methods

#### Determination of PAH concentration in mussel samples

The following PAHs; Naphthalene (Naph), acenaphthylene (Acy), acenaphthene (Ace), flourene (Flo), phenanthrene (Phe), anthracene (Ant), fluoranthene (Flu), pyrene (Pyr), chrysene (Chy), benzo[g,h, i]perylene (BghiP), dibenzo[a,h]anthracene (DahA), Indeno[1,2,3-cd]pyrene (IcdP), benzo[a]pyrene (BaP), benzo[k]fluoranthene (BkF), benzo[b]fluoranthene (BbF) and benzo[a]anthracene (BaA) were estimated in two species of mussels *Donax trunculus* and *Andara duloii*. PAH extraction was performed as follows: freshly collected mussel samples from each species were dissected and the soft tissue from 30 to 40 individual bivalves was pooled together and dried in an open oven at 50 °C. Five grams of the dried samples were extracted in methanol with a Soxhlet extractor for 8 h followed by a lipid saponification step with the addition of potassium hydroxide and distilled water to the mixture and Soxhlet reflux extraction was continued for additional 2 h. Then, the methanolic part was extracted 3 times with n-hexane. The n-hexane fraction extracts were dried with anhydrous sodium sulfate and concentrated under a rotary evaporator. Subsequentially, concentrated samples were cleaned and fractioned on a chromatographic column (silica gel, aluminum oxide, and anhydrous sodium sulfate) and finally sample elution from the column was performed by adding an n-hexane to elute saturated aliphatic fraction (F1) and a mixture of n-hexane and dichloromethane (9:1) to elute hydrocarbon fraction (F2). Nitrogen gas was used to concentrate the samples and 2 µl of each sample and PAH standards were subjected to GC–MS analysis. The response factor of the individual PAH compounds to the internal standard was measured and calculated at least 3 times in the beginning, in the middle, and at the end for each batch of GC injections (10 samples). The method detection limits for each PAH compound ranged from 0.3 to 1.1 ng g^ − 1^ of wet weight. According to previous reports [18], the extracted PAHs were identified and quantified using Gas chromatography, (Agilent technologies 1200 series), at the National Institute of Oceanography &Fisheries, Alexandria. The 16 priority PAHs were identified and quantified by comparing their retention time with a mixture of PAH standards.

### Biochemical analysis

#### Determination of plasma catalase enzyme activity

Catalase enzyme activity was determined by the spectrophotometric method [19]. Briefly, 50 µl of the sample was mixed with 0.5 ml of phosphate buffer and 0.1 ml of hydrogen peroxide and, incubated for one minute at 37 °C. Then chromogen inhibitor and peroxidase enzymes were added. The mixture was incubated for 10 min at 37 °C. Sample blank, standard blank, and standard samples were performed for quality control. All samples were measured at 510 nm by spectrophotometer. The analysis principle relies on the Catalase enzyme reacting with a known quantity of H_2_O_2_, and the reaction was stopped with a catalase inhibitor after one minute.
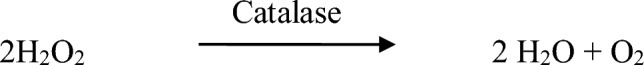


The remaining H_2_O_2_ reacts with 3,5-dichloro-2-hydroxybenzene sulfonic acid (DHBS) and 4-aminophenazone (AAP) in the presence of hydrogen peroxide (HRP) to form a colored complex whose absorbance is inversely proportional to the concentration of catalase in the sample.
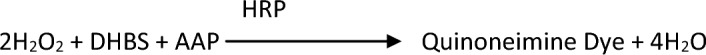


Catalase activity was determined according to the following equation.

$${\text{Catalase activity in plasma (U/L)}}\,\,{ = }\,\,\,\,\frac{{\text{A standard - A sample}}}{{\text{A standard}}}\,\,\,*1000$$

#### Determination of erythrocyte glutathione-S-transferase (GST)

The Biodiagnostic assay test kit estimates total GST activity (cytosolic and microsomal) by measuring the conjugation of 1-chloro-2,4-dinitrobenzene (CDNB) with GSH. The conjugation is associated with increased absorbance at 340 nm, with the rate of increase in direct proportion to the GST activity in the sample [20]. Briefly: 50 µl of the sample was mixed with phosphate buffer (1 ml), and GSH (0.1 ml) and incubated for 5 min at 37 °C. Then, CDNB was added, and the mixture was incubated for 5 min at 37 °C. Then, the reaction was terminated by adding trichloroacetic acid. Finally, the sample was centrifuged at 3000 rpm for 5 min and the absorbance of the sample was measured at 340 nm against blank. GST activity was calculated according to the following equation.

$${\text{Enzyme activity}}\,\,\, = \frac{{A_{340} \,{\text{sample}}}}{0.0096}\,\times \,\frac{{{\text{Total Volume}}\,\,{(1}{\text{.35)}}}}{{{\text{Volume of Sample (0}}{.05)}}}$$

Where: 0.0096 is the molar extinction coefficient of CDNB at 340 nm.

#### Estimation of malondialdehyde (MDA)

Estimation of MDA was done using the thiobarbituric acid (TBA) assay. MDA formed from the breakdown of polyunsaturated fatty acid serves as a convenient index for determining the extent of the peroxidation products in the body. TBA reacts with MDA in an acidic medium at a temperature of 95 °C for 30 min to form the TBA reactive product. The absorbance of the resultant pink product can be measured at 534 nm. MDA in the sample react with TBA to give a red colored (MDA-TBA) whose absorbance was measured at 534 nm. Briefly, TBA was added to 200 µl of the serum and the reaction mixture was heated to 95 °C for 30 min in a temperature-controlled heating block. The reaction was stopped by placing the reaction mixture on ice after which the concentration of MDA in the sample was read at 534 nm [21]. MDA concentration was measured according to the following equation:

$${\text{Malondialdhyde (MDA) concentration in serum (nmol/ml) }}\,\,{ = }\,\,\frac{{{\text{A }}\,{\text{sample}}}}{{{\text{A }}\,{\text{standard}}}}\,\times\,\,10$$

where: 10, concentration of standard.

#### Estimation of reduced glutathione (GSH)

The modified standard Ellman’s method was employed in the estimation of GSH in which, the tested serum (100 µl) was added to Ellman’s reagent (5,5’-dithiobis-2-nitrobenzoic acid (DNTB)). The reduced chromogen is directly proportional to GSH concentration, and its absorbance can be measured at 405 nm [22]. GSH concentration was calculated according to the following equation:

$${\text{Blood glutathione (mg/dl)}}\,\, = \,\,\frac{{A_{sample} \, \times 30}}{0.45}\, = \,A_{sample} \times\,\,66.66$$

where: 30 is the concentration of standard and 0.45 is the absorbance of standard.

### Determination of serum Progesterone (P4) hormone

Serum P4 estimation was done using the enzyme-linked immunosorbent assay method which is based on the principle of the specific interaction between antigens and their corresponding antibodies. The absorbance of the color complex formed after a competitive reaction between biotinylated antibody, native antigen, and enzyme-antigen conjugate for a limited number of antibody binding sites is proportional to the concentration of P4 in the sample [23]. Briefly, microplates’ wells for each serum reference, control, and patient specimen were formatted to be assayed in duplicate. 25 µl of the sample was pipetted into the provided microwell plate from the manufacturer’s ELISA kit, then, 50 µl of P4 enzyme was added and the plate was gently steered for 10–20 s. Progesterone biotin reagent was added to all wells and the plate was gently mixed by stirring for additional 10 s. The plate was incubated for 60 min at room temperature. Then The contents of the microplate were discarded by decantation or aspiration followed by a washing step with a washing buffer three times. Then the substrate solution was added to each well and incubated for 20 min at room temperature, and the reaction was terminated by adding a stop solution. Finally, the absorbance of all samples was measured at 450 nm in a microplate reader. The concentration for each unknown was obtained from the standard curve.

### Determination of serum follicle stimulating hormone (FSH)

Estimation of FSH utilizes the principle of specific interaction between antigens and their corresponding antibodies The absorbance of the color complex formed after a competitive reaction between biotinylated antibody, native antigen, and enzyme-antigen conjugate for a limited number of antibody binding sites is proportional to the concentration of FSH in the sample [24]. Briefly, microplates' wells for each serum reference, control, and patient specimen were formatted to be assayed in duplicate. 25 µl of the sample was pipetted into the provided microwell plate from the manufacturer’s ELISA kit, then, 100 µl of FSH enzyme was added and the plate was gently steered for 10–20 s. The plate was incubated for 60 min at room temperature. Then the contents of the microplate were discarded by decantation or aspiration followed by a washing step with a washing buffer three times. Then the substrate solution was added to each well and incubated for 15 min at room temperature, and the reaction was terminated by adding a stop solution. Finally, a microplate reader measured the absorbance of all samples at 450 nm (using a reference wavelength of 620–630 nm to minimize well imperfections).

### Determination of serum BPDE-albumin adduct

BPDE-albumin adduct measurement was carried out by immunoassay detection method. Bovine serum albumin (BSA) standards and samples were added into 96 wells of ELISA plates and incubated at 37 ℃ for 2 h. Anti-BPDE-1 antibody was used to probe for the BPDE-albumin adducts present in the standard and sample, followed by horse radish peroxide (HRP) conjugated secondary antibody. The concentration of BPDE-albumin in the sample is determined from a standard curve prepared from predetermined BPDE-BSA standards [25]. Briefly, 100 µl of the sample or reduced/BPDE-BSA standards were added to the 96-well albumin binding plate. All were incubated at 37 ºC for at least 2 h or 4 °C overnight. Wells were washed with PBS three times and then wells were taped on an absorbent pad. Then the diluted anti-BPDE-I antibody was added to all wells and incubated for 1 h at room temperature on an orbital shaker followed by adding the diluted secondary antibody-HRP conjugate to the wells. Substrate solution was added and incubated for 2–3 min. Finally, the reaction was stopped by adding a stop solution, and each well’s absorbance was measured at 450 nm in a microplate reader. The reduced BSA standard was used as an absorbance blank.

### Determination of 1-naphthol and 2-naphthol

The estimation of 1-naphthol and 2-naphthol was performed by high-performance liquid chromatography (HPLC). Briefly: Urine samples were enzymatically hydrolyzed with 30 µl of β-glucuronidase and sulfatase for 16 h at 37 ℃ in a shaking water bath. After hydrolysis, 5 ml of acetonitrile was added and mixed for 10 s. The samples were centrifuged at 1000 rpm for 10 min. A 20 µl of supernatant was injected into the HPLC (Agilent technologies 1200 series). The mobile phase used was acetonitrile–water (35:65%) + 100 µl acetic acid per liter solvent, at a flow rate of 1 ml/min. the excitation and emission wavelengths were 227 nm and 430 nm respectively and the fluorescence detector. The column used was Eclipse XDB-C18 (Made in the USA) 150 mm X 4.5 mm. Quantification of the 1-naphthol and 2-naphthol in the urine sample is obtained by standard curve by measuring different concentrations of the reference standard, and then the curve plotted by instrument data analysis program and the final concentration of the 1-naphthol in the urine sample expressed as µmol/mol creatinine from the following equation:

$$\begin{aligned}& {\text{Conc}}{\text{. of 1 - naphthol and 2 - naphthol (}}\mu {\text{mol/mol creatinine)}}\\&\quad = \,\,\frac{{\text{Conc of Sample}}}{{\text{Creatinine conc}}}\,\times \,\frac{1}{144.17} \end{aligned}$$

where: 144.17 is molecular weight of α-naphthol and β-naphthol.

### Determination of urine creatinine

Estimation of urine creatinine was done using a modified Jaffe’s reaction method. Principally, creatinine in the sample reacts with alkaline picrate within a specific time interval to avoid interferences to form a colored complex whose absorbance is proportional to the concentration of creatinine in the sample [26]. Briefly, diluted urine samples were mixed with alkaline picrate and the absorbance (A1) after 30 s and after 120 s (A2) of the sample addition was measured at 492 nm. Creatinine concentration was calculated according to the following equation:

$$\Delta {\text{A}} = {\text{A}}2 - {\text{A}}1$$

$$\begin{aligned} {\text{Creatinine (mol/l)}} &= \,\,\frac{{\Delta {\text{A}}\,{\text{sample}}}}{{\Delta {\text{A}}\,{\text{standard}}}}\\&\quad \times \,{\text{Standard concentration}}\, \times \,50 \end{aligned}$$

where: 50 is the dilution factor.

### Statistical analysis

Data were analyzed using IBM SPSS software package version 20.0. The Shapiro–Wilk test and the D’Agstino test were used to test for normality in the distribution of quantitative variables. Vision test was done with histogram and QQ plot. Quantitative data were described using mean and standard deviation for normally distributed data while abnormally distributed data were expressed using median, minimum, and maximum. For normally distributed data, analysis of variance and post hoc tests were used to determine variations among multiple groups’ means. For abnormally distributed data, Mann–Whitney and Kruskal Wallis test were used to compare multiple groups’ means. The spearman test was used to study the correlation between different parameters. The significance of the obtained results was judged at the 5% level.

## Results

### Mussels PAH content

**Fig. 1:**
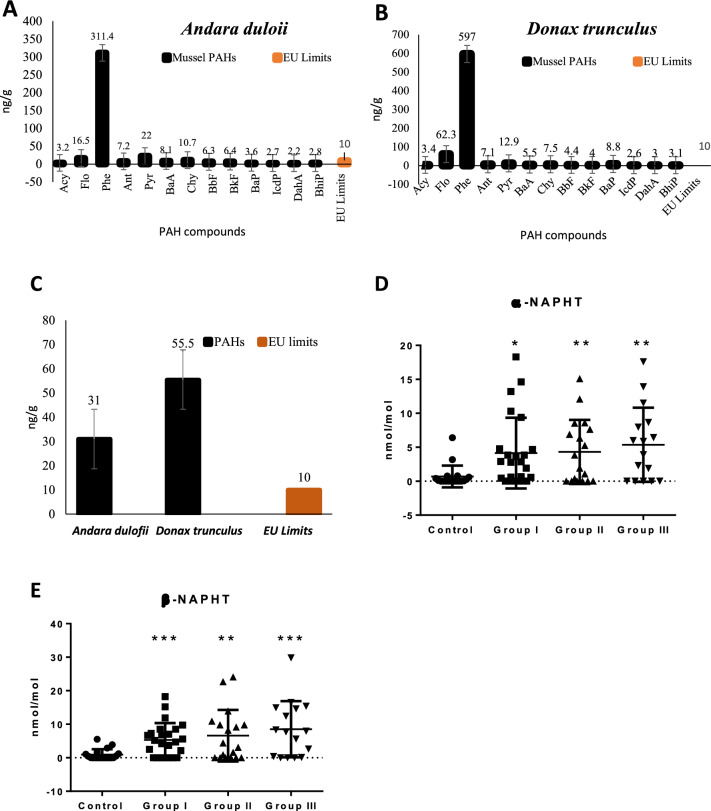
Poly Aromatic Hydrocarbon (PAH) concentration in different mussel species of (**A**) *Andara duloii* and (**B**) *Donax truculus.* A comparison between PAH levels in mussel species and their European limit is shown in figure (**C**). Figures (**D**), (**E**) shows PAH metabolite concentration in different pregnant women groups (as control vs abortion groups I-III). The control group comprises 18 normal, healthy, and fertile women, group I comprises 24 women with 2 medically unexplained recurrent spontaneous abortions, group II comprises 18 women with 3 medically unexplained recurrent spontaneous abortions and group III comprises 16 women with more than 3 medically unexplained recurrent spontaneous abortions. Data points for each group are represented by circles, squares, triangles, and inverted triangles, and the mean, as well as standard deviation lines, are presented in the background for each group. Statistical significance at *p*-value < 0.05. Significant different at *p* < 0.05 *, *p* ≤ 0.01**, and *p* ≤  0.001***

As can be seen in Fig. 1A, the occurrence of the PAHs in *Andara duloii* is presented. The levels of Flo, Phe, Pyr, and Chy in *Andara duloii* were above the EU maximum limits with Phe having the highest concentration. The levels of other PAHs were below the maximum limits. Figure 1B shows the occurrence of the PAHs in *Donax trunculus*. The levels of Flo, Phe, and Pyr in *Donax trunculus* were above the EU maximum limits with Phe having the highest concentration. The levels of other PAHs were below the maximum limits. As can be seen in Fig. 1C, the mean concentration of PAHs in *Donax trunculus and Andara duloii* is presented. The mean levels of PAHs in the two species of mussels were above the EU maximum limits indicating their contamination with PAHs. Finally, analysis of PAH metabolites α and β-naphtol were higher in recurrent pregnancy loss women in comparison to the control group (Fig. 1D, E).

### Biochemical indices in women without recurrent pregnancy loss (RPL) and women with RPL

**Fig. 2:**
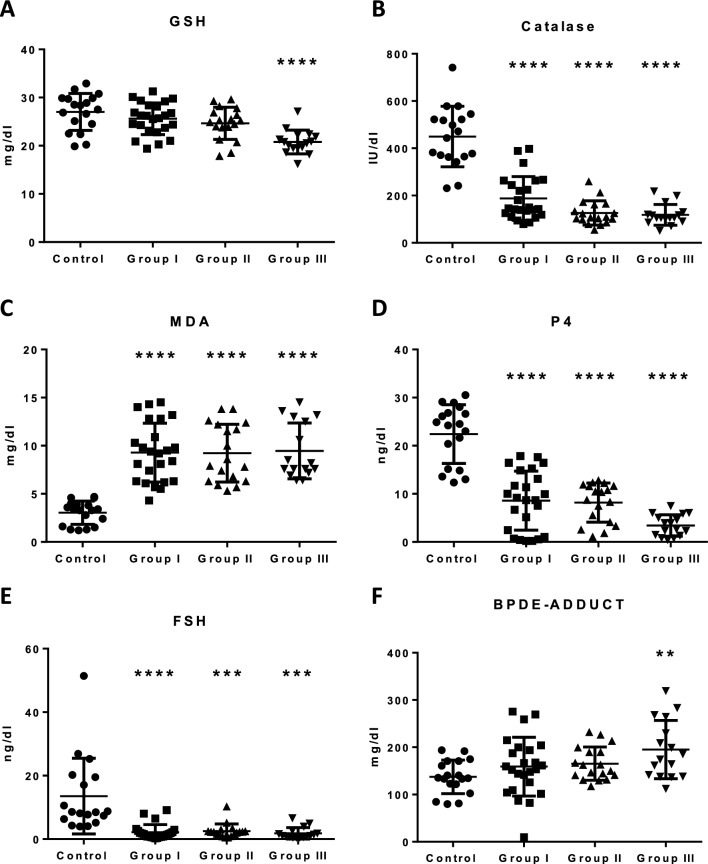
Impact of increasing levels of PAHs metabolites in different women groups on their levels of oxidative stress markers of GSH (**A**), Catalase (**B**), and MDA (**C**). Progesterone (P4) serum concentration is shown in (**D**), reproductive hormones FSH in (**E**), and BPDE-albumin adduct formation in different women groups (control vs groups I-III) is shown in (**F**). The control group comprises 18 normal, healthy, and fertile women, group I comprises 24 women with 2 medically unexplained recurrent spontaneous abortions, group II comprises 18 women with 3 medically unexplained recurrent spontaneous abortions and group III comprises 16 women with more than 3 medically unexplained recurrent spontaneous abortions. Data points for each group are represented by circles, squares, triangles, and inverted triangles, and the mean, as well as standard deviation lines, are presented in the background for each group. Statistical significance at *p*-value < 0.05. Significant different at *p* < 0.05 *, *p* ≤ 0.01**, *p* ≤ 0.001*** and *p* ≤ 0.0001 ****

**Fig. 3:**
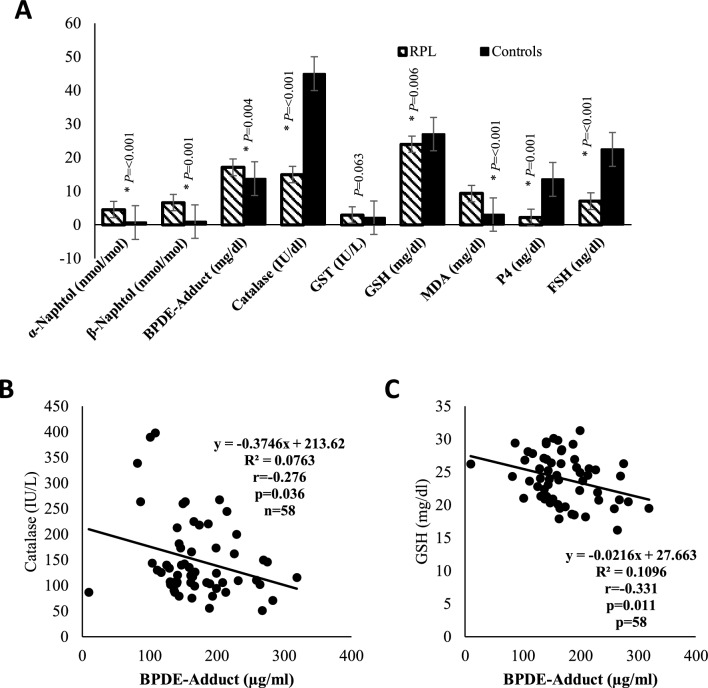
Impact of elevated levels of PAH metabolites on different physiological factors in different women groups (control vs groups I-III) (**A**) and the correlation between catalase and BPDE-Adduct is shown in (**B**) and GSH and BPD-adduct is shown in (**C**). The spearman test was used to analyze the correlation between each pair of parameters. Data are presented as mean ± standard deviation in Fig. 3A. Statistical significance at *p*-value < 0.05. Significant different at *p* < 0.05 *, *p* ≤ 0.01**, and *p* ≤ 0.001***

To precisely get a clear insights into the actual underlined mechanism of the association of PAH accumulation in women and records of their miscarriage. We started to analyze several factors in women’s sera related to oxidative stress status, antioxidant defense activity, P4 sex hormone level, and formation of albumin adduct. Our results have shown that recurrent aborted women (groups I-III) showed lower levels of GSH (Fig. 2A), Catalase (Fig. 2B), P4 (Fig. 2D), and FSH (Fig. 2E). additionally, women with successive abortions showed higher levels of the 10-epoxide-albumin adduct (Fig. 2F) and MDA (Fig. 2C). The comparison of urinary PAH metabolites, indices of oxidative stress, and reproductive hormones in women without recurrent pregnancy loss (RPL) (Control) and women with RPL (Groups I-III) were depicted in Fig. 3A and (Table 1). Higher levels of BPDE-albumin, MDA, α and β-naphthol and lower GSH, catalase, FSH, and P4 were observed in women with RPL (Groups I-III) compared to control (*p* =  < 0.001).

**Table 1:** Comparison of PAH metabolites, indices of oxidative stress and reproductive hormones in women without recurrent pregnancy loss (RPL) (Group I) and women with RPL (Groups II-IV)

Index	Control n = 18	Group I n = 24	Group II n = 18	Group III n = 16	*P*-value	Control vs Grp I *P*-value	Control vs Grp II *P*-value	Control vs Grp III *P*-value
α-Naphtol (nmol/mol)	0.69 ± 1.61	4.13 ± 5.20	4.32 ± 4.72	5.37 ± 5.45	0.026*a	0.007b	0.031b	0.015b
β-Naphtol (nmol/mol)	0.96 ± 1.59	5.33 ± 5.01	6.59 ± 7.68	8.52 ± 8.39	0.008*a	0.004b	0.031b	0.003b
BPDE-Adduct (µg/ml)	137.37 ± 35.47	159.08 ± 62.10	165.31 ± 35.19	195.24 ± 61.66	0.030*a	0.134	0.047b	0.003b
Catalase (IU/L)	449.70 ± 128.08	187.67 ± 92.66	126.35 ± 51.38	118.52 ± 43.96	< 0.001*a	< 0.001b	< 0.001b	< 0.001b
GST (IU/L)	2.11 ± 0.87	2.97 ± 4.16	2.53 ± 1.03	3.21 ± 1.12	0.022*a	0.868	0.047b	0.003b
GSH (mg/dl)	27.02 ± 3.85	25.60 ± 3.28	24.65 ± 3.35	20.78 ± 2.47	< 0.001*a	0.166	0.214	< 0.001b
MDA (mg/dl)	3.04 ± 1.21	9.28 ± 3.06	9.22 ± 3.00	9.46 ± 2.90	< 0.001*a	< 0.001b	< 0.001b	< 0.001b
P_4_ (ng/dl)	13.54 ± 11.91	2.18 ± 2.36	2.50 ± 2.24	1.83 ± 1.80	< 0.001*a	< 0.001b	< 0.001b	< 0.001b
FSH (ng/dl)	22.43 ± 6.09	8.59 ± 6.12	8.18 ± 4.06	3.43 ± 2.23	< 0.001*a	< 0.001b	< 0.001b	< 0.001b

Data presented as mean ± SD**,** * = indicates significant variations among the groups at *p *< 0.05

a = *p* values from Kruskal Wallis test

b = *p* values from Mann–Whitney U test

*GST* glutathione-s-transferase, *GSH* reduced glutathione, *MDA* malondialdehyde, *P4* progesterone, *FSH* follicle stimulating hormone

### Associations between benzo[a]pyren-7,8-dihydrodiol-9,10-epoxide-albumin adduct and antioxidant enzymes.

Spearman correlation analysis test showed that the relationship between PAH-albumin adduct with antioxidant enzymes in women with RPL is negatively associated with catalase (r = − 0.276, *p* = 0.036), and GSH (r = − 0.331, *p* = − 0.011) only in women with RPL (Fig. 3B, C).

## Discussion

Recurrent pregnancy loss has been attributed to various factors including genetic abnormalities, endocrine dysfunctions, maternal disorders, microbial infections, and chemical toxicity arising from exposure to environmental pollutants including PAH. PAH pollutants have been associated with adverse health conditions including reproductive toxicity probably arising from PAH-induced disruption of the endocrine homeostasis or redox imbalance [27]. Here in the current research, the indices of oxidative stress, PAH protein adduct, and metabolites about exposure to PAH-contaminated food were assessed in women with RPL.

In this study, although we did not provide a direct link between the elevated levels of PAHs in the two studied mussel species and the recurrent pregnancy loss reported in different women groups (I, II, and III). From our point of view, we found that it is important to give a piece of preliminary information on the current environmental levels of these pollutants in the Mediterranean Sea of Alexandria in geographical areas close to their residence localities. In addition, as part of the common dietary habits of seafood consumption of the Egyptian population, we found that it is important to collect two of the common edible mussel species and measure their PAH contents. Surprisingly, the mean concentration of PAHs in the two mussel species was higher than the EU safe limits. A previous study has also reported the increased concentrations of many PAHs along the Mediterranean coast of Alexandria which may pose a great health risk to the population [28]. On the other hand, PAH concentration was relatively low when measured in another mussel *Brachidontes Sp* collected from the Red sea coast of Egypt [29] indicating that aquatic pollution is changing dramatically over the years and can varies between geographical locations. Another study reported low to moderate concentration levels of PAH in mussel *Mytilus* *galloprovincialis* collected from aquaculture farms along the Mediterranean coast of Greece [3]. These different results not only indicated that different mussel species can be used as an environmentally relevant bio-indicator of PAH pollution and concentration in the aquatic environment but also that consumption of PAH-enriched mussel species poses a significant health risk to humans.

Regarding particulate air matter, a study published in 2020, reported that PAHs concentration in particulate air samples collected during the summer season in Alexandria, Egypt showed a high concentration of Phe, Flo, and Pyr among other PAH compounds than that during winter, Autumn, and Spring seasons (Zahran et al. 2020). On the other hand, another study indicated that BaP, BkF, and Indeno were the highest PAHs in atmospheric suspended particulate matter samples collected from urban, suburban, and rural sites at Alexandria [30]. Also, it was reported that other cities in Egypt constitute high levels of PAHs in their atmosphere. Analysis of particulate air samples in Cairo, Egypt showed that their concentration during the winter season is higher than that of the summer [31]. In Cairo, levels of PAHs in atmospheric suspended particulate matter vary in their concentration between different collection sites in Cairo, Egypt which can be below or higher than WHO guidelines [32]. According to [33] levels of flu and phe were among the highest PAH component in Assiut air in Egypt. These reported findings may indicate the contribution of non-vehicular sources of PAH contamination and PAH pollution as possible exposure and a health risk factor for humans.

Taken individually, only Phe, Fluo, and Pye were above the safe limits with Phe having the highest concentration in the two studied species of mussel. Higher levels of Phe in mussels may be related to their higher water solubility. The distribution of PAHs including phenanthrene in mussels is a function of their solubility in water, which indicates their absorption in water dissolved form [34]. Mussels are filter-feeding bivalves and are susceptible to pollutants that are either soluble in water or adsorbed on the filtered particles [35]. PAH levels in mussel tissues have been correlated with the concentration of PAH in sediments and the octanol/water partition coefficient (Kow) [36, 37].

Moreover, we observed a higher levels of urine α and β-naphthol in women with RPL compared to the control group. Urine metabolites of naphthalene; 1-naphthol and 2-naphthol have commonly used as a markers of naphthalene exposure with 2-naphthol being a more specific marker as 1-naphthol is also present in carbaryl pesticides [38, 39]. One of the earliest studies have indicated that naphthalene exposure led to a pregnancy abortion dated back to 1973 [40]. A previous study has demonstrated higher levels of 2-hydroxy naphthalene, 1-hydroxy naphthalene, and 1-hydroxy pyrene in the urine of infertile subjects compared to their control counterparts [41]. Higher urinary concentrations of PAH metabolites have also been demonstrated in patients suffering from early pregnancy loss indicating the possible adverse health impacts of high levels of environmental PAHs on the reproductive outcome [42]. Furthermore, naphthalene metabolites were reported in newborn babies of exposed mothers through dietary ingestion of mothballs [43]. The observed positive association between PAH levels and early pregnancy loss suggests that the urinary concentration of its metabolites might be used as an early predictive risk marker in miscarried patients exposed to high PAH levels [44]. It is commonly accepted that PAH metabolite level in the urine is frequently used as an indicator of PAH exposure via different routes (inhalation, dermal absorption, and ingestion) [45]. The presence of α and β-naphthol in both women with RPL and controls indicates that both groups were exposed to PAH. However, the systemic PAH levels in different individuals are a function of the dose, duration of exposure, and variations in the inherent genetic peculiarities associated with PAH metabolizing enzymes. The inter-individual differences which exist in levels of expression and catalytic activities of these metabolizing enzymes in humans are responsible for individual differences in response to PAH exposures and consequently individual PAH levels, PAH metabolites, and PAH adduct formation so observed [46]. This may explain the disparities between urine α and β-naphthol levels in both groups of women.

Our study demonstrated a higher level of BPDE-albumin in women with RPL (Groups I-III) compared to the control group. Higher levels of BPDE protein adducts have been reported in women exposed to cigarette smoke group compared to control ones [47]. Apoptosis of human trophoblasts has been associated with higher BPDE concentration. Chorion explant migration from women who had undergone elective abortion is inhibited by BPDE in a concentration dependent manner [9, 10]. Higher levels of PAH DNA adduct have been reported in the placenta of pregnant women with preterm delivery than in those with full-term delivery [11, 12, 48]. Accumulation of BPDE-DNA adducts in the placenta or BPDE across the placenta barrier has been associated with toxicity to fetal development [11, 12]. Individual peculiarities in response to PAH exposure may be responsible for the disparities in the BPDE-albumin levels observed in both groups [46].

Lower levels of FSH and P4 were observed in women with RPL compared to controls. In line with our findings, lower mean FSH values have been demonstrated in women with RPL when compared to the control group [49]. Lower levels of P4 have also been documented in women with RPL compared to their control counterparts in a previous study [50]. Lower FSH and P4 levels observed in women with RPL in this study may be linked to the high PAH levels observed in this group as indicated by higher levels of PAH protein adduct (BPDE-albumin) and urine PAH metabolites (α and β-naphthol). Previous in vitro studies have shown that exposure to PAH has been associated with diminished levels of plasma hormones, P4, estrogen, prolactin, and fetal survival [51]. PAHs have been shown to alter P4 receptor expression and secretion of P4 and estradiol from placental and granulosa cell lines [14, 52]. The proposed pathway underlying PAH-induced disruption of reproductive hormone homeostasis may involve its binding and stimulation to the aryl hydrocarbon receptor (AhR), formation of DNA adducts and reactive metabolites leading to the disruption of the hypothalamic-gonadal axis, depression of steroidogenic enzyme activities and impaired trophic hormone stimulation [45]. Lower P4 levels have been associated with compromised integrity of the decidualized endometrium resulting in disruption of the implantation site which may result in a recurrent miscarriage of unexplained etiology. P4 has also shown to create a conducive environment for the embryo during implantation by stimulating morphological and physiological changes in the endometrium [50].

Our study observed lower GSH and catalase activity with higher MDA and GST activity in women with RPL compared to controls. Previous studies have also demonstrated decreased selenium levels, catalase and glutathione peroxidase activities, and increased MDA and lipid peroxides in serum and/or placental tissues of recurrent miscarriage patients [53, 54]. Significantly elevated levels of MDA with decreasing trend of GSH were found in women in the preterm delivery group than in women in a full-term delivery group [7]. Increased expression of GST protein has been reported in women with unexplained pregnancy loss compared to controls [55]. The high GST level in women with RPL suggests the presence of a state of increased demand to oppose the effects of oxidative stress in such patients [56]. Again, perturbations in oxidant/antioxidant balance observed in women with RPL may be attributed to higher PAH levels as evidenced by higher levels of PAH protein adducts and urine PAH metabolites as observed in these women. Owing to the hydrophilic nature of PAHs, they are directly pass-through mammalian cell membranes and are then metabolically activated by cytoplasmic enzymes to electrophilic quinones and semiquinones [57]. These reactive intermediates can undergo redox cycling to generate large amounts of free radical species that can upset the redox balance leading to oxidative stress [38, 39]. Exposure to PAHs has been associated with increased levels of indices of oxidative stress and inflammation and adverse pregnancy outcomes among pregnant women by previous studies [58, 59]. B[a]P has been linked with redox imbalance and oxidative stress [60]. It has been implicated in the reduction in the activities of antioxidant enzymes such as catalase, GST, and SOD, decreased levels of antioxidant molecules such as vitamin C, E, and GSH, increased lipid peroxidation, and expression of proinflammatory cytokines [9, 10].

Negative associations were also observed between BPDE-albumin and catalase and GSH only in women with RPL. BPDE-albumin has been shown to induce the increased generation of ROS, depletion of antioxidants, and consequently oxidative stress [11, 12]. [BPDE 2, BPDE]. This relationship may explain the observed negative association between the BPDE-albumin adduct and the antioxidants. Mechanisms involving impaired placental development or degeneration of syncytiotrophoblast in early pregnancy have been implicated in oxidative stress-induced pregnancy-related disorders [61].

Our finding on the association between high levels of different PAHS in recurrent pregnancy loss women and control women can be summarized as follows: Briefly, high PAHs were associated with 10-epoxide-albumin adduct formation and MDA levels, on the other hand, they reduced level of GSH, catalase, P4, and FSH. Those findings indicate that PAH can exert different physiological parameters leading to a high level of abortion in women.

Our study is limited by the small sample size and the single spot sampling method. Because of the short half-life of PAH metabolites, single spot sampling may not precisely classify the duration of exposure to PAH, However, this shortcoming was compensated for by the BPDE-albumin estimation.

## Data Availability

The data will be available upon request and after the manuscript is published.

## Abbreviations

Ace: Acenaphthene
Acy: Acenaphthylene
Ant: Anthracene
BaA: Benzo[a]anthracene
BaP: Benzo[a]pyrene
BbF: Benzo[b]fluoranthene
BghiP: Benzo[g,h,i]perylene
BkF: Benzo[k]fluoranthene
BPDE-albumin: Benzo[a]pyren-7,8-dihydrodiol-9,10-epoxide-albumin adduct
Chy: Chrysene
DahA: Dibenzo[a,h]anthracene
Dl: Deciliter
ER: Estrogen receptor
Flo: Fluorene
Flu: Fluoranthene
FSH: Follicle stimulating hormone
GC: Gas chromatography
GSH: Reduced glutathione
GST: Glutathione-S-Transferase
HPLC: High-performance liquid chromatography
IcdP: Indeno[1,2,3-cd]pyrene
IU: International unite
MDA: Malondialdehyde
Naph: Naphthalene
ng: Nanogram
nmol: Nanomole
OS: Oxidative Stress
P4: Progesterone
PAH: Poly aromatic hydrocarbon
Phe: Phenanthrene
Pyr: Pyrene
ROS: Reactive oxygen species
RPL: Recurrent pregnancy loss
µg: Microgram
